# Response of *Atalantia buxifolia* to Salt Stress Based on Physiological and Transcriptome Analysis

**DOI:** 10.3390/biology15010065

**Published:** 2025-12-30

**Authors:** Yujie Yang, Wenxu Hu, Jianmiao Chen, Jinwang Qu, Cheng Chen, Chu Wu

**Affiliations:** College of Horticulture & Gardening, Yangtze University, Jingzhou 434025, China; yjyang@yangtzeu.edu.cn (Y.Y.); 2023720949@yangtzeu.edu.cn (W.H.);

**Keywords:** germination, seedling, salt stress, *Atalantia buxifolia*, physiological and biochemical analysis, transcriptome analysis

## Abstract

As a coastal brush, *Atalantia buxifolia* is a good rootstock of citrus plants around sea shores, but its salt tolerance has not been studied. In order to explore the salt tolerance of *A. buxifolia*, its seeds and seedlings were subjected to NaCl stress treatment, followed by phenotypic observation and biochemical and transcriptome analysis. Results showed that the increase in NaCl concentrations resulted in the decrease in germination rates, germination potential, germination index, and vigor index of *A. buxifolia* seeds, as well as growth of epicotyl and radicle, and biomass of *A. buxifolia* seedlings. However, the seeds of *A. buxifolia* could adapt to the growth of 100 mM NaCl concentration to a certain extent. The levels of malondialdehyde (MDA) and relative electrolyte leakage increased with the increase in NaCl concentrations. However, under treatment of 100 mM NaCl, the biomass, POD, CAT, APX, GSH, AsA, H_2_O_2_, MDA, and relative electrolyte leakage of *A. buxifolia* seedlings did not show significant changes compared with the control treatment. Transcriptome analysis showed that expression of differential genes increased with the increase in NaCl concentrations. GO enrichment showed that the most annotated genes were metabolic process, cell and cell composition, and binding. The KEGG pathway annotation shows that differential genes were mainly enriched in some pathways, such as photosynthesis antenna proteins, plant hormone signal transduction, glutathione metabolism, and starch and sucrose metabolism. In addition, differentially expressed genes had been annotated into 45 transcription factor families, including the largest number of bHLH, NAC, WRKY, MYB, and bZIP families. The results provide a basis for further understanding the salt tolerance mechanism and exploring related salt tolerance genes of *A. buxifolia.*

## Simple Summary

To elucidate the salt tolerance of the coastal citrus rootstock *Atalantia buxifolia*, we analyzed its physiological and transcriptomic responses to NaCl. While increasing salt concentrations suppressed germination and growth, seeds exhibited partial tolerance at 100 mM NaCl. Furthermore, transcriptome analysis identified salt-induced genes primarily involved in metabolic and signaling pathways such as plant hormone transduction and antioxidant metabolism. Notably, many differentially expressed genes encoded transcription factors from the bHLH, NAC, and WRKY families. These findings offer a foundation for isolating salt-tolerance genes from this species.

## 1. Introduction

Soil salinization leads to land degradation, which has become an increasingly serious global environmental problem under the background of global climate change, hindering agricultural production. The total area of salinized soil in the world is over 3.6 × 109 hm^2^ [1]. About 100 cities in China are located in saline-alkali areas, and about 3.3 × 105 hm^2^ of agricultural land was reduced or abandoned due to soil salinization [2]. The widely distributed saline-alkali soil not only causes a decrease in agricultural land resources and deteriorates the ecological environment [3], but also seriously affects plant growth and crop yield [4]. How to use and change the existing saline and alkali soil, taking into account the ecological and economic interests, is crucial to achieve sustainable agricultural development, especially under the background of population increase around the world.

Sodium chloride (NaCl) is the most common salt in soil that causes damage to plant growth and development [5]. Previous studies showed that salt stress mainly affects plant physiological and biochemical processes in three aspects, i.e., osmotic stress, ion toxicity, and nutritional imbalance. Excessive ion accumulation in salinized soil affects infiltration, resulting in a decrease in soil water potential, thereby inhibiting water absorption by the plant roots, inducing stomatal closure and reducing gas exchange, furthermore decreasing photosynthesis rates in plants [6]. In addition, excessive ion absorption by plant roots causes ion toxicity, which leads to excessive production of reactive oxygen species (ROS), such as hydrogen peroxide (H_2_O_2_), hydroxyl radicals (OH·), singlet oxygen (^1^O_2_), and superoxide anions (O_2_^−^) [7]. The ROS accumulation in plants under salt stress causes damage of plant cells, such as increase in membrane permeability, protein oxidation, membrane lipid peroxidation, and enzyme inactivation [8]. In order to decrease the damage caused by salt stress, plants have evolved physiological adaptation strategies, such as antioxidant systems, osmotic adjustment, ion homeostasis, and hormone regulation. The plants overcome oxidative damage through activating the antioxidant system [9]. Antioxidant enzymes include peroxidase (POD), catase (CAT), superoxide dismutase (SOD), glutathione reductase (GR), and ascorbate peroxide (APX). At the same time, non-enzymatic systems include some antioxidants, such as ascorbic acid (AsA), glutathione (GSH), and α-tocopherol [10]. In addition, osmoregulatory substances, such as soluble proteins and sugars, betaine, and proline can maintain cell osmotic balance and protect cell structures and functions under salt stress [11]. It was found that the *IhCHS1* gene is induced to express under salt stress, significantly improving salt tolerance in *Arabidopsis* seedlings in the mature stage [12]. A novel MYB transcription factor, *VhMYB60*, was successfully isolated and cloned from the ‘Beta’ grapevine. It was found that the expression of *VhMYB60* was significantly upregulated in response to salt and cold stress in both mature leaves and grape roots. Furthermore, *Arabidopsis* plants overexpressing *VhMYB60* exhibited markedly enhanced tolerance to salt and cold stress, as well as increased survival rates, compared to control [13].

*Atalantia buxifolia* (Poir.) Oliv., a member of the subfamily Aurantioideae within the family Rutaceae, is an evergreen shrub or small tree that can reach up to 2.5 m in height; This species is widely recognized as a medicinal plant and is predominantly distributed in subtropical and tropical regions in Asia, including China, Sri Lanka, southern India, and eastern Bangladesh. *A. buxifolia* typically thrives in coastal areas as well as inland regions with acidic soils, showing notable salt tolerance [14]. Previous studies have isolated and identified various secondary metabolites from this plant species, including terpenoids (such as tetraterpenoids and sesquiterpenoids), acridones, and coumarins [15]. Due to its salt-tolerant properties, *A. buxifolia* is commonly used as a rootstock to enhance the salt tolerance of citrus cultivars [16]. However, the mechanisms underlying its salt tolerance have not been thoroughly investigated. To facilitate the use of *A. buxifolia* as a rootstock for citrus cultivars in saline soils, it is essential to study its salt tolerance mechanisms and identify novel genes associated with salt-stress adaptation in this species.

In this study, the seeds and seedlings of *A. buxifolia* were treated with different NaCl concentrations, and the salt tolerance of the plants was investigated on the morphological and physiological levels. In addition, transcriptome analysis was carried out to understand the expression of related genes in leaves of *A. buxifolia* seedlings under salt stress.

## 2. Materials and Methods

### 2.1. Plant Culture and Salt Treatment

#### 2.1.1. Seed Germination of *A. buxifolia*

The black mature fruits of *A. buxifolia* were obtained in the botanical garden in the western campus of Yangtze University (30°21′17″ N, 112°8′18″ E). The fruit flesh was removed and the seeds were collected. The healthy seeds with the same sizes were selected and rinsed using tap water for 2 h. The seed coat was removed, followed by immersion in 75% ethanol for 30 s, and disinfection with 2% sodium hypochlorite for 14 min. Then, the sterilized seeds were rinsed three times using sterilized distilled water and placed in 1/2 MT culture medium in test tubes (2.8 cm in diameter and 15 cm in length) containing 0, 100, 200, and 300 mM NaCl, respectively. These sterilized seeds were sown on MS (Murashige and Skoog). The control was treated with distilled water. Each tube contained 10 mL the culture medium, pH 5.9, one seed in each test tube and 25 test tubes for a treatment group, three biological repeats for each treatment group. The seeds were cultured in the growth chamber under 25 °C in the dark.

#### 2.1.2. Culture of *A. buxifolia* Seedlings

Seeds were sterilized as mentioned above. Then the sterilized seeds were evenly sown in a rectangular plastic pots (19 cm × 14 cm × 5 cm) filled with clean vermiculite. The transparent plastic cover was opened after the obvious epicotyl. One month later, they were transplanted to a plastic pot (diameter 6.5 cm, height 7 cm) with seedling substrate (Yimu Agricultural Technology Co., Ltd., Beijing, China), 2 plants per pot. After 2 months of plant growth, the seedlings with consistent growth were randomly selected for salt-stress treatment. A total of four treatments were set up, 40 pots per treatment, and watered every 5 days with different Hoagland nutrient solutions containing 0, 100, 200, 300 mM NaCl, 50 mL per pot, watered 4 times, and the bottom of the pots with trays. The experimental materials were cultured in growth room at 25 °C under a 16/8 h light/dark photoperiod. After 21 days, the treated plants were collected. After liquid nitrogen treatment, the materials were stored at −80 °C for subsequent experiments.

### 2.2. Experimental Methods

#### 2.2.1. Analysis of Seed Germination of *A. buxifolia* Under NaCl Stress

Seeds were sown for 0 days (d), and germination was observed and recorded daily. On the 21st day, the lengths of the epicotyl and hypocotyl were measured using a vernier caliper and ruler. Additionally, five seedlings were randomly selected to determine their fresh weight (FW). Then, they were deactivated at 105 °C for 30 min and dried to constant weight at 70 °C, and the dry weight (DW) was weighed. The germination rate, germination potential, germination index, salt tolerance suitable concentration, half lethal concentration, limited concentration, and relative water content (RWC) of the seeds were calculated [17,18].

Germination percentage (%) = (Number of germinated seeds in 21 d/total number of tested seeds) × 100%

Germination potential = number of seeds germinated on the 7th day/total number of tested seeds × 100%

Germination index (GI) = ∑Gt/Dt

In the formula, Gt is the germination number of different time (d); Dt is the corresponding germination time.

Vigor index = GI × S

In the formula, S is seedling length (cm).

Relative salt damage rate = (CK germination rate − treatment group germination rate)/CK germination rate × 100%

RWC (%) = FW/DW × 100%

#### 2.2.2. Transcriptome Analysis of *A. buxifolia* Seedlings in Response to Salt Stress

According to the effect of salt stress on seedlings, three levels of NaCl concentration were set up, i.e., 0, 100, and 200 mM. The salt treatment method was carried out as mentioned above. After 21 days of salt treatment, the mature leaves of *A. buxifolia* were collected. After liquid nitrogen treatment, the transcriptome sequencing was performed by Beijing Nuohe Zhiyuan Technology Co., Ltd. (Beijing, China) In this analysis, the samples CK, J, and B represented the three treatments, respectively. The three replicates of CK1, CK2, and CK3 were obtained under 0 mM treatment, the three replicates of J1, J2, and J3 obtained under 100 mM treatment, and the three replicates of B1, B2, and B3 obtained under 200 mM treatment.

#### 2.2.3. Determination of Gas Exchange

Salt-stress treatment for about 14 days, performed in sunlight at 9: 00–12: 00 in the morning, randomly selected 5 pots, select 5 healthy leaves, using Yaxin-1102 portable photosynthetic analysis system (Beijing Yaxin Liyi Technology Co., Ltd., Beijing, China) for gas exchange determination. Before determination, the plants were exposed to direct sunlight for 15 min.

#### 2.2.4. Chlorophyll Fluorescence Determination

After 14 days of salt-stress treatment, 5 pots were randomly selected, and 4 healthy leaves were cut from each pot. The chlorophyll fluorescence was measured by FluorCam system (Photo System Instruments, Prague, Czech Republic) chlorophyll fluorescence imaging system. The leaves were dark treated for 15 min before measurement.

#### 2.2.5. Determination of Morphological Indexes

After 21 days, the morphological indexes were measured. The ground diameter and plant height were measured by vernier caliper and ruler. The roots were scanned and analyzed by the system of Epson scanner (EPSON scan v3.771, Nagano Prefecture, Japan) series plant root image analyzer, including root length, root tip number, root surface area, and average diameter. Five strains of each treatment were randomly selected to weigh the fresh weight (FW), then killed at 105 °C, dried to constant weight at 70 °C, and weighed at the dry weight (DW), and then the relative water content (RWC) was calculated (as shown in the Section 2.2.1).

#### 2.2.6. Determination of Physiological Parameters

The activity of superoxide dismutase (SOD) was measured by the nitrogen blue tetrazolium colorimetric method [19]. The activity of peroxidase (POD) was measured by guaiacol colorimetric method of Chen et al. [20]. The activity of catalase (CAT) and glutathione reductase (GR) was detected by Solarbio kit (Beijing Solarbio Science, Beijing, China). Ascorbate peroxidase (APX) was measured according to the method of Subramanyam et al. [21]. The concentrations of ascorbic acid (AsA) were determined according to the method of Nejati-Yazdinejad et al. [22] and Majidi et al. [23]. The concentrations of glutathione (GSH) were determined using the dithionitrobenzoic acid (DTNB) method [24]. The concentrations of hydrogen peroxide (H_2_O_2_) were determined by potassium iodide colorimetric method [25]. The concentrations of malondialdehyde (MDA) were determined by the thiobarbituric acid (TBA) method according to Fazeli et al. [26]. The relative conductivity was determined by the immersion method [27]. Proline was determined using ninhydrin colorimetry [28], levels of soluble sugars were determined by anthrone colorimetry [29], levels of soluble proteins were determined by Coomassie brilliant blue G-250 colorimetry [30], and concentrations of photosynthetic pigments were determined by the Kumar ethanol method [31].

### 2.3. RNA-Seq Technology Process

#### 2.3.1. RNA Extraction and Qualification, and Library Preparation for Transcriptome Sequencing

RNA was extracted from the leaves of *A. buxifolia* by the standard extraction method. RNA integrity was assessed using the RNA Nano 6000 Assay Kit of the Bioanalyzer 2100 system (Agilent Technologies, Santa Clara, CA, USA). Total RNA was used as input material for the RNA sample preparations. Then PCR was performed with Phusion High-Fidelity DNA polymerase, Universal PCR primers and Index (X) Primer. At last, PCR products were purified (AMPure XP system) and library quality was assessed on the Agilent Bioanalyzer 2100 system.

#### 2.3.2. Clustering and Sequencing (Novogene Experimental Department)

The clustering of the index-coded samples was performed on a cBot Cluster Generation System using TruSeq PE Cluster Kit v3-cBot-HS (Illumia, San Diego, CA, USA) according to the manufacturer’s instructions. After cluster generation, the library preparations were sequenced on an Illumina Novaseq platform and 150 bp paired-end reads were generated. The data has been uploaded, the datasets generated and analyzed during the current study are available in the [Mendeley data] repository, [https://data.mendeley.com/datasets/4zpjtzfpxr/1 (accessed on 1 October 2025)].

#### 2.3.3. Quality Control

Raw data (raw reads) of fastq format were firstly processed through fastp software (v0.23.x). By removing reads containing adapter, ploy-N, and low-quality reads from raw data, clean reads were obtained. At the same time, Q20, Q30, and GC content of the clean data were calculated. All the downstream analyses were based on the clean data with high quality.

#### 2.3.4. Reads Mapping to the Reference Genome

Reference genome and gene model annotation files were downloaded from the genome website (http://citrus.hzau.edu.cn/download.php, accessed on 1 October 2025) (MA, USA) directly. Index of the reference genome was built using Hisat2 (v2.0.5) and paired-end clean reads were aligned to the reference genome using Hisat2 (v2.0.5). Mismatch tolerance during Hisat2 alignment was set using the parameters. Mapping mode refers to the number and types of alignment locations permitted for a single sequencing read by Hisat2. In this project, only uniquely mapped reads were retained for downstream analysis.

#### 2.3.5. Quantification of Gene Expression Level and Differential Expression Analysis

FeatureCountsv1.5.0-p3 was used to count the reads numbers mapped to each gene. Then, the FPKM of each gene was calculated based on the length of the gene and reads count mapped to this gene. During data quality control, reads containing adapter sequences were removed, reads with undetermined bases (N) were discarded, and low-quality reads were filtered out based on the criterion that more than 50% of the bases in a read had a Phred quality score (Qphred) ≤ 5. Differential expression analysis of two groups was performed using the DESeq2R package (1.20.0). The resulting *p*-values were adjusted using the Benjamini and Hochberg’s approach for controlling the false discovery rate. Genes with an adjusted *p*-value ≤ 0.05 found by DESeq2 were assigned as differentially expressed.

#### 2.3.6. GO and KEGG Enrichment Analysis of DEGs and Gene Set Enrichment Analysis

Gene Ontology (GO) enrichment analysis of differentially expressed genes (DEGs) was implemented by the ClusterProfiler (3.8.1) package. GO terms with corrected *p* value < 0.05 were considered significantly enriched by DEGs. We used cluster Profiler (3.8.1) package to test the statistical enrichment of DEGs in KEGG pathways. Gene set enrichment analysis can include subtle expression changes. We used the local version of the GSEA analysis tool (http://www.broadinstitute.org/gsea/index.jsp, accessed on 1 October 2025) (MA, USA), GO, KEGG data set were used for GSEA independently.

### 2.4. Data Analysis

The experimental data were used to organize and calculate. In this paper, the data were subjected to analysis of variance and analysis of significance using SPSS 20.0 (IBM, Chicago, IL, USA), and graphs were plotted with Sigmaplot 14.0. Different letters were used to represent significant differences as determined by Duncan’s multiple range tests (*p* < 0.05).

## 3. Results

### 3.1. Effect of Salt Stress on Seed Germination of A. buxifolia

**Figure 1:**
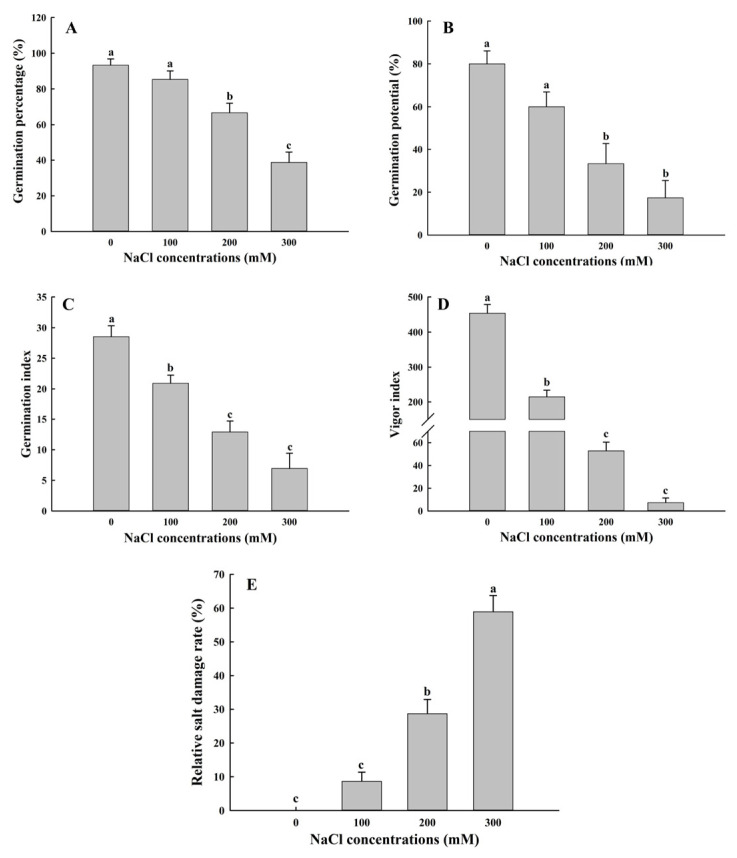
Effects of different NaCl concentrations on the germination and relative salt damage rate of *A. buxifolia* seeds. (**A**) Germination percentage; (**B**) germination potential; (**C**) germination index; (**D**) vigor index; (**E**) relative salt damage. Different lowercase letters denote significant differences (*p* < 0.05), and the same applies below.

The germination rate, germination potential, germination index, and vigor index of *A. buxifolia* seeds decreased with the increase in NaCl concentrations (Figure 1). The germination rate began to decrease significantly from 100 mM NaCl treatment (*p* < 0.05, Figure 1A), while the germination rate under 100 mM NaCl treatment decreased by only 8%, compared with control, showing no significant difference. The germination rates under 200 and 300 mM NaCl treatment were significantly lower than that under 0 and 100 mM NaCl treatment (*p* < 0.05), and compared with control, the germination rate under 200 and 300 mM NaCl, respectively, decreased by 26.67% and 54.67%. However, the germination rate of *A. buxifolia* seeds reached 66.7% under 200 mM NaCl treatment (Figure 1A). Compared with control, the germination potential under 100 mM NaCl decreased by 20%, but the difference was not significant between the two treatments (*p* > 0.05, Figure 1B). The germination potential under 100 mM NaCl and control was significantly higher than those under 200 and 300 mM NaCl treatment (*p* < 0.05, Figure 1B), but there was no significant difference between 200 and 300 mM NaCl treatments (*p* > 0.05, Figure 1B). The results showed that when the NaCl concentration reached 100 mM, the germination rate and germination potential of the seeds did not significantly change in two treatments (0 and 100 mM NaCl), indicating that the salt concentration had no obvious inhibitory effect on the germination of *A. buxifolia* seeds. In terms of germination index and vigor index (Figure 1C,D), the overall trend of these two parameters was consistent. The two parameters under control were significantly higher than the other three treatments (*p* < 0.05, Figure 1C,D), and those under 100 mM NaCl were also significantly higher than 200 and 300 mM NaCl (*p* < 0.05, Figure 1C,D), and there was no significant difference between 200 and 300 mM NaCl (*p* > 0.05, Figure 1C,D). When NaCl concentration reached 300 mM, the germination rate, germination potential, germination index, and vigor index of the seeds were 41.4%, 21.7%, 24.3%, and 1.6% of control, respectively (Figure 1), indicating that seed germination under 300 mM was inhibited greatly. However, under 200 and 300 mM NaCl, the seeds of *A. buxifolia* still had certain vitality (Figure 1), indicating that the seeds of *A. buxifolia* showed salt tolerance to some extent.

In addition, as shown in Figure 1E, with the increase in salt concentration, the relative salt injury rates of *A. buxifolia* seeds increased, and the injury rates under 300 mM concentration were significantly higher than those under the other three treatments (*p* < 0.05, Figure 1E). However, the 100 mM treatment and control showed no significant change, indicating that 100 mM NaCl treatment had a lower salt injury rate of *A. buxifolia* seeds.

### 3.2. Effects of Salt Stress on the Growth of A. buxifolia Seedings After Germination

**Figure 2:**
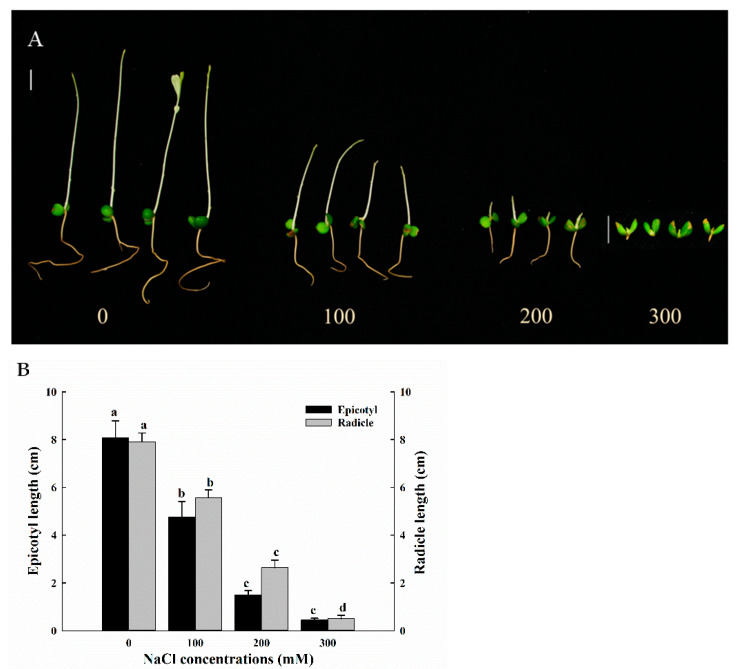
Effects of salt stress on the growth of *A. buxifolia* seedlings after germination. (**A**) Effects of different NaCl concentrations on growth of the germination characteristics of *A. buxifolia* seeds (Bar = 1 cm). (**B**) Effects of salt-stress treatment on length of embryonic axis of *A. buxifolia* seeds at germination stage (0: mM NaCl; 100: 100mM NaCl; 200: mM NaCl; 300: mM NaCl; The same as below).

Salt stress had an inhibitory effect on the growth of *A. buxifolia* seedlings after germination. With the increase in NaCl concentration, the effect on the epicotyl and radicle of *A. buxifolia* seedlings was more significant (Figure 2A). Under NaCl 300 mM, the epicotyl grew and the radicle inhibits growing (Figure 2A). The epicotyl growth under control was significantly higher than the other three concentrations (*p* < 0.05, Figure 2A,B). The epicotyl length under 100, 200, and 300 mM NaCl treatments were 58.7%, 18.4%, and 5.5% of control, respectively, significantly lower than those under control (*p* < 0.05, Figure 2B). The epicotyl length under 100 mM NaCl was significantly higher, compared with 200 and 300 mM NaCl (*p* < 0.05, Figure 2B). However, there was no significant difference between 200 and 300 mM treatments in the epicotyl length (*p* > 0.05, Figure 2B). However, there was a significant difference in radicles between 200 and 300 mM NaCl treatment (*p* < 0.05, Figure 2B). With the increase in NaCl concentrations, the growth of radicle of *A. buxifolia* seeds showed a decreasing trend. Basically, the length difference between each continuous treatment was about 2–3 cm, and the length between the four treatments was significantly different (Figure 2B, *p* < 0.05).

**Figure 3:**
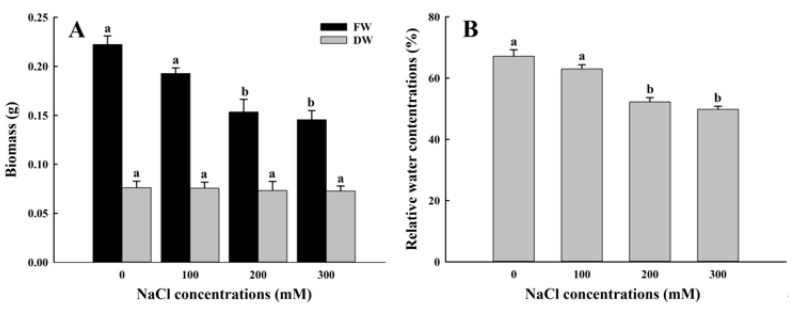
Effects of different NaCl concentrations on growth of biomass of *A. buxifolia* seedlings. (**A**) Biomass; (**B**) relative water concentrations.

Under salt stress, with the increase in NaCl concentrations, the growth fresh weight, dry weight, and relative water concentrations of *A. buxifolia* seedlings showed a decreasing trend (Figure 3). Compared with control and 100 mM NaCl treatment, the fresh weight under 200 and 300 mM treatments was significantly lower (*p* < 0.05, Figure 3A), and there were no significant differences in fresh weight between 100 mM NaCl and control and between 100 and 200 mM treatment (*p* > 0.05, Figure 3A). Then, there were no significant differences in dry weight among the four concentrations (*p*> 0.05, Figure 3A). In addition, there was no significant difference in the relative water concentrations of seedlings between 0 and 100 mM NaCl treatment (*p* > 0.05, Figure 3B), but the relative water concentrations under 0 and 100 mM NaCl treatment were significantly higher than those under 200 mM and 300 mM NaCl treatment (*p* < 0.05, Figure 3B), while no significant differences in relative water concentrations were found between 200 and 300 mM NaCl (*p* > 0.05, Figure 3B).

With the increase in NaCl concentration, the yellowing symptoms of *A. buxifolia* leaves appeared gradually, and significant differences in leaves were noted among NaCl concentrations. Under the treatment of 300 mM NaCl, the drying symptom appeared from the edge of the leaves to the whole leaves, and this situation appeared in the middle and upper leaves of the plant, and then developed downward until the whole plant withered (Figure S1).

**Figure 4:**
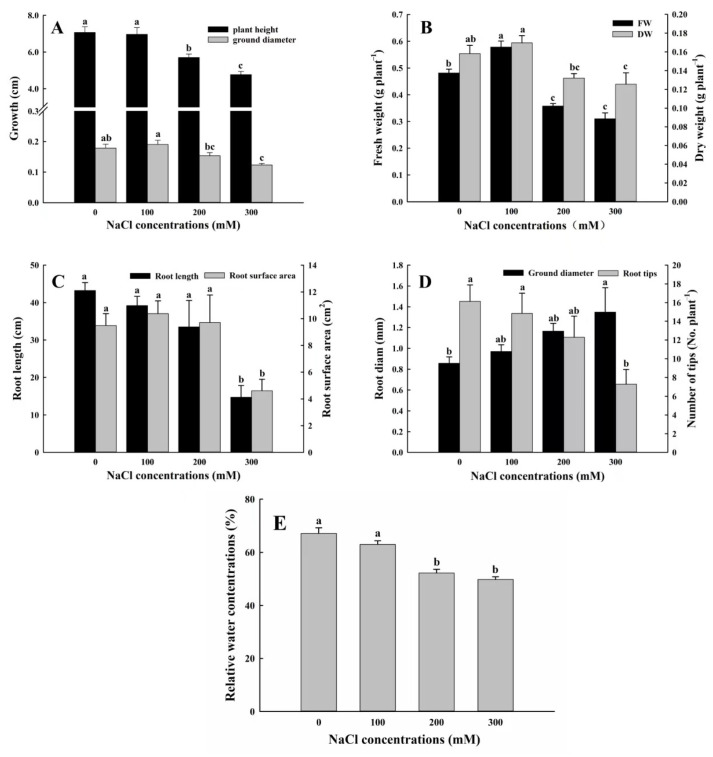
Effects of different NaCl concentrations on growth of *A. buxifolia* seedlings. (**A**) Growth; (**B**) fresh weight and dry weight; (**C**) root length and root surface area; (**D**) root diameter and root tips; (**E**) relative water content.

The plant height of *A. buxifolia* seedlings decreased with the increase in NaCl concentrations (Figure 4A). The plant height under 100 mM NaCl treatment, accounting for 98.6% of control, there was no significant difference in plant height among the two treatments (*p* > 0.05, Figure 4A). The plant height under 0 mM and 100 mM NaCl treatment was significantly higher than those under treatments of 200 mM and 300 mM NaCl (*p* < 0.05, Figure 4A). Furthermore, the greatest ground diameter reached 0.18 cm under 100 mM NaCl, while there was no significant difference between 100 mM NaCl and control (*p* > 0.05, Figure 4A). However, under 100 mM NaCl, ground diameters were significantly higher than those under 200 mM and 300 mM (*p* < 0.05, Figure 4A). The ground diameters under control were not significantly different from those under 200 mM NaCl (*p* > 0.05, Figure 4A), but significantly greater than those under 300 mM NaCl treatment (*p* < 0.05, Figure 4A).

As shown in Figure 4B, the fresh and dry weight reached the highest under 100 mM treatment and were significantly higher than those under 200 mM and 300 mM treatment (*p* < 0.05, Figure 4B), but there were no significant differences in dry weight between control and 100 mM NaCl treatment (*p* > 0.05, Figure 4B).

From 0 to 300 mM NaCl concentration, the root length and root tip numbers decreased, and there was no significant change in the number of these two indexes among the other three treatments except for 300 mM NaCl treatment (*p* > 0.05, Figure 4C,D). The root length of the other three treatments was significantly higher than that under 300 mM treatment (*p* < 0.05, Figure 4C). Compared with the treatments of 0 and 100 mM NaCl treatment, the root tip numbers of seedlings under 300 mM NaCl treatment were significantly decreased (*p* < 0.05, Figure 4D). Then, the root surface area reached the maximum under 100 mM NaCl treatment, and the root surface areas were significantly lower under 300 mM NaCl treatment, compared with the other three treatments (*p* < 0.05, Figure 4C). With the increase in NaCl concentration, average ground diameters showed an increasing trend. Among them, the average ground diameters of seedlings under 300 mM NaCl treatment were significantly higher the control group (*p* < 0.05, Figure 4D). In general, with the increase in salt concentration, the root growth of *A. buxifolia* seedling was inhibited, and the root length and root tip number decreased, while the increase in salt concentration expanded the surface area and diameter of the roots of *A. buxifolia* seedlings. However, there was no significant change in these indexes of seedlings at 100 mM and 200 mM (*p* > 0.05, Figure 4C,D), indicating that there was no significant effect on the root growth of *A. buxifolia* seedlings under a range of salt concentrations.

The relative water content of *A. buxifolia* seedlings was the highest under 100 mM NaCl treatment and the lowest under 300 mM NaCl treatment (Figure 4E). Furthermore, the relative water content under 100 mM NaCl treatment was significantly higher than those under 200 and 300 mM NaCl treatment (*p* < 0.05, Figure 4E), but there was no significant change in relative water content between 100 mM treatment and control, suggesting that some extent of salt stress can promote relative water content in the leaves of *A. buxifolia* seedlings.

### 3.3. Effect of Salt Stress on Gas Exchange of A. buxifolia Seedlings

**Figure 5:**
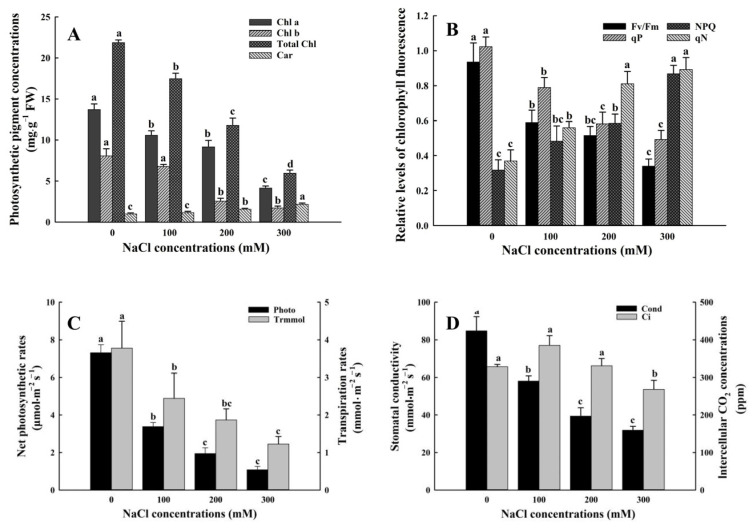
Effect of salt stress on gas exchange of *A. buxifolia* seedlings. (**A**) Photosynthetic pigment concentrations; (**B**) chlorophyll fluorescence parameters; (**C**) net photosynthetic rates and transpiration rates; (**D**) stomatal conductance and intercellular CO_2_ concentrations.

Chlorophyll a (Chl a), chlorophyll b (Chl b) and total chlorophyll content decreased with increasing salt concentration, while carotenoids increased (Figure 5). The contents of chlorophyll a under control were significantly higher than those under the other three treatments (*p* < 0.05, Figure 5A), and levels of Chl a under 100 mM treatment was also significantly higher than that under 300 mM treatment (*p* < 0.05, Figure 5A), but there was no significant difference in Chl a between 100 mM and 200 mM treatment. The chlorophyll b levels under 0 mM and 100 mM treatment were significantly higher than those under 200 mM and 300 mM treatment (*p* < 0.05, Figure 5A). In terms of total chlorophyll content, there were significant differences among the four treatments (*p* < 0.05, Figure 5A). There was no significant difference in carotenoids between 0 mM and 100 mM treatment, but the carotenoids under 300 mM treatment was significantly higher than those under the other three treatments (*p* < 0.05, Figure 5A).

The maximum photochemical efficiency (*Fv*/*Fm*) and photochemical quenching coefficient (*qP*) of photosystem II (PSII) decreased with increase in NaCl concentration, and *Fv*/*Fm* and *qP* under control was significantly higher than those under the other three treatments (*p* < 0.05). *Fv*/*Fm* did not change significantly between 100 mM and 200 mM treatment. However, compared with the 100 mM treatment, *Fv*/*Fm* under 300 mM treatment decreased significantly (*p* < 0.05, Figure 5B). *qP* under 200 mM treatment was significantly lower, compared with 100 mM treatment. The non-photochemical quenching coefficient (*qN*) of variable fluorescence shows an upward trend with the increase in salt concentration. Non-photochemical fluorescence quenching (*NPQ*) under 300 mM treatment was significantly higher than those under the other three treatments (*p* < 0.05, Figure 5B). There was no significant difference in *qP* between 200 mM and 300 mM treatments, but qP under 200 mM and 300 mM treatment were significantly higher than the other two treatments (*p* < 0.05, Figure 5B). In addition, there was a significant difference in *qP* between control and 100 mM treatment (Figure 5B, *p* < 0.05).

As shown in Figure 5, the net photosynthetic rate (Photo), transpiration rate (Trmmol) and stomatal conductance (Cond) decreased with the increase in salt concentration, and the three gas exchange parameters under control was significantly higher than those under the other three treatments (*p* < 0.05, Figure 5C,D). The levels of Photo and Cond under 100 mM treatment were significantly higher than those under 200 mM and 300 mM treatment (*p* < 0.05, Figure 5C,D). Levels of Trmmol under 100 mM treatment were significantly higher than those under 300 mM treatment (*p* < 0.05, Figure 5C), while there was no significant difference in Trmmol between 100 mM and 200 mM treatment. Intercellular CO_2_ concentrations (Ci) reached the highest under 100 mM NaCl treatment, but there was no significant difference among 0 mM, 100 mM NaCl, and 200 mM NaCl. However, Ci concentrations under 300 mM treatment were significantly lower than those under the other three treatments (*p* < 0.05, Figure 5D).

### 3.4. Effects of Salt Stress on Antioxidant System of A. buxifolia Seedlings

#### Activities of Antioxidant Enzymes and Levels of Antioxidants

**Figure 6:**
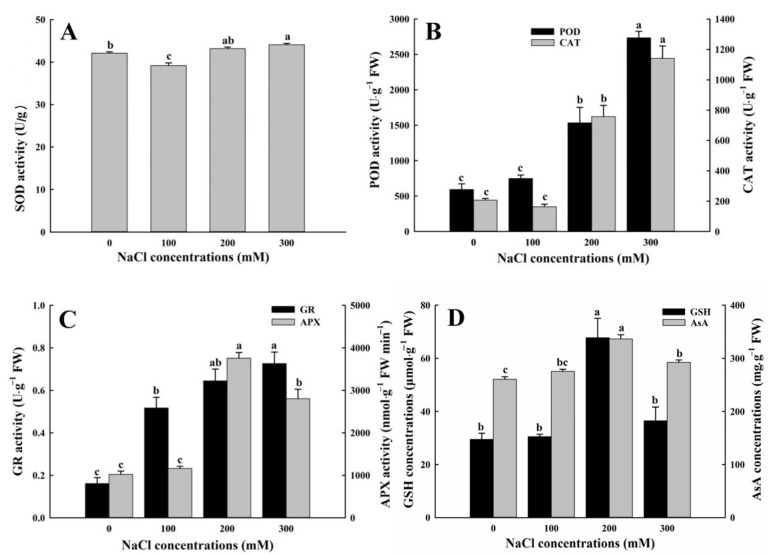
Effects of different NaCl concentrations on activities of antioxidant enzymes and levels of antioxidants in leaves of *A. buxifolia* seedlings. (**A**) Activities SOD; (**B**) activities of POD and CAT; (**C**) activities of GR and APX; (**D**) levels of GSH and AsA.

SOD (superoxide dismutase) activities under 100 mM NaCl treatment were significantly lower than the other three treatments (*p* < 0.05, Figure 6A), and SOD activities under 0 mM NaCl treatment were significantly lower than that under 300 mM treatment (*p* < 0.05), while there was no significant difference in SOD activities between 0 and 200 mM NaCl treatment (Figure 6A). CAT (catalase) activities were the lowest under 100 mM NaCl treatment, 21.1% lower than that under control, but there was no significant difference between the two treatments (*p* > 0.05, Figure 6B). CAT activities under 200 mM and 300 mM NaCl treatment were significantly higher than those under 0 and 100 mM NaCl treatment (*p* < 0.05, Figure 6B). CAT activities under 200 and 300 mM NaCl treatment were 265.9% and 450.1% higher than those under control, respectively (Figure 6B). POD (peroxidase) activities under 100 mM NaCl treatment were higher than those under control (Figure 6B), but were significantly lower than those under 200 and 300 mM NaCl treatment (*p* < 0.05, Figure 6B), while POD activities under 200 mM NaCl treatment were significantly lower than those under 300 mM NaCl treatment (*p* < 0.05, Figure 6B).

As shown in Figure 6C, the activities of ascorbate peroxidase (APX) increased first and then decreased with increase in NaCl concentrations, and reached the highest at 200 mM NaCl treatment, and APX activities under 200 mM NaCl treatment were significantly higher than those under the other three treatments (*p* < 0.05, Figure 6C). However, compared with control, APX activities under 100 mM NaCl treatment was only increased by 13.6%, no significant between them (*p* > 0.05, Figure 6C). APX activities under 200 and 300 mM NaCl treatment were significantly higher than those under 0 and 100 mM NaCl treatment (*p* < 0.05, Figure 6C). In addition, activities of glutathione reductase (GR) increased with increase in NaCl concentrations, and the activities under 300 mM NaCl treatment were significantly higher than those under control and 100 mM NaCl treatment (*p* < 0.05, Figure 6C). GR activities under control were significantly lower than those under the other three treatments (*p* < 0.05, Figure 6C). Although GR (glutathione reductase) activities under 200 mM NaCl treatment were higher than those under 100 mM NaCl treatment, there was no significant difference between the two treatments (*p* < 0.05, Figure 6C).

The levels of ascorbate (AsA) and glutathione (GSH) reached the highest values under 200 mM NaCl treatment, and their levels under this treatment were significantly higher than those under the other three NaCl treatments (*p* < 0.05, Figure 6D). Furthermore, compared with control, AsA levels under 300 mM NaCl treatment were significantly lower (*p* < 0.05, Figure 6D), but there was no significant difference in AsA levels between control and 100 mM NaCl treatment (*p* > 0.05, Figure 6D). In addition, except for 200 mM treatment, GSH levels showed the following pattern: 300 mM > 100 mM > 0 mM, but there were no significant differences between the three treatments.

### 3.5. Changes in Levels of Osmotic Adjustment Substances of A. buxifolia Seedlings Under Salt Stress

**Figure 7:**
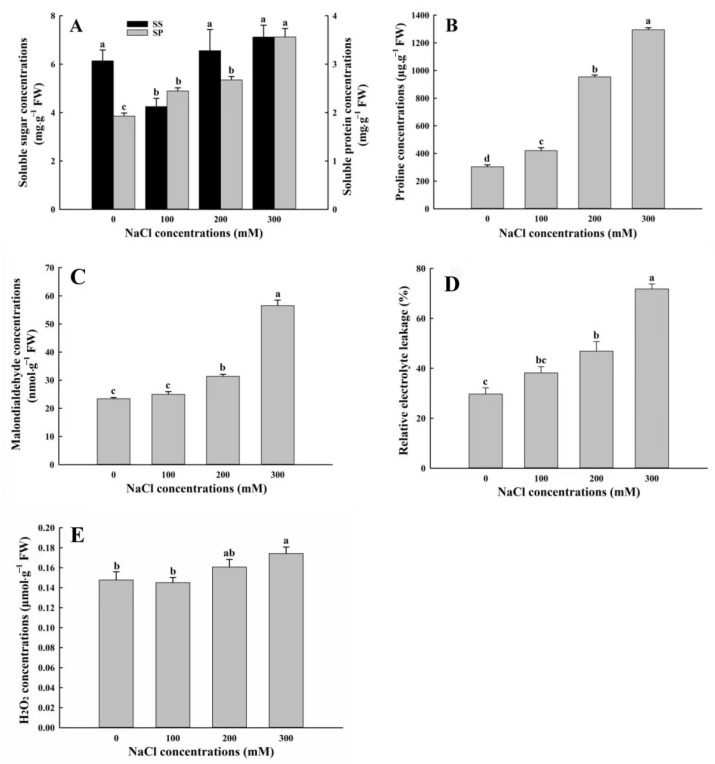
Effects of different NaCl concentrations on osmotic adjustment substances in leaves of *A. buxifolia* seedlings. (**A**) Levels of soluble sugars and proteins; (**B**) levels of proline; (**C**) levels of MAD; (**D**) levels of relative electrolyte leakage; (**E**) levels of H_2_O_2_.

Changes in soluble protein (SP) levels showed the following pattern: 300 mM > 200 mM >100 mM > 0 mM, and SP levels under 300 mM NaCl treatment were significantly higher than those under the other three treatments (*p* < 0.05, Figure 7A). There was no significant difference in SP levels between 100 and 200 mM NaCl treatment, but their SP levels were significantly higher than those under control (*p* < 0.05, Figure 7A). Furthermore, the levels of soluble sugar (SS) were the highest under 300 mM NaCl treatment and the lowest under 100 mM NaCl treatment, and SS levels under 100 mM treatment were significantly lower than those under the other three treatments (*p* < 0.05, Figure 7A). In addition, proline levels increased gradually with the increase in NaCl concentrations. There were significant differences in proline levels among the four treatments (*p* < 0.05, Figure 7B).

### 3.6. Effects of Salt Stress on Malondialdehyde, Relative Electrolyte Leakage, and Hydrogen Peroxide of A. buxifolia Seedlings

The levels of malondialdehyde (MDA) under 300 mM NaCl stress were significantly higher, compared with the other three treatments (*p* < 0.05, Figure 7C). Secondly, when the NaCl concentration was 200 mM, MDA levels were significantly higher than those under 0 and 100 mM NaCl treatment (*p* < 0.05, Figure 7C). MDA levels were not significantly different between control and 100 mM NaCl treatment (*p* > 0.05, Figure 7C). The relative electrolyte leakage showed such change pattern: 300 mM > 200 mM > 100 mM > 0 mM with the increase in NaCl concentrations. There was no significant difference in relative electrolyte leakage between 200 mM and 100 mM treatment. Levels of relative electrolyte leakage under 300 mM treatment were significantly higher than those under the other three treatments (*p* < 0.05, Figure 7C). Levels of the relative electrolyte leakage under 100 mM treatment were 8.5% higher than under control (Figure 7D).

With the increase in NaCl concentrations, the levels of hydrogen peroxide (H_2_O_2_) showed the following pattern: 300 mM > 200 mM > 0 mM > 100 mM. H_2_O_2_ (hydrogen peroxide) levels under 300 mM NaCl treatment were significantly higher than those under control and 100 mM NaCl treatment (*p* < 0.05, Figure 7E). Furthermore, there were no significant differences in H_2_O_2_ levels among control, 100 and 200 mM NaCl treatment (*p* > 0.05, Figure 7E).

### 3.7. Transcriptomic Analysis

#### 3.7.1. Outline of Transcriptional Analysis

To investigate the effects of salt stress on transcriptional changes, we carried out RNA sequencing on leaves of *A. buxifolia* seedlings treated with 0, 100, and 200 mM NaCl. A total of 59.3 Gb clean bases were obtained in mRNA sequencing for 9 samples with at least 5.93 Gb for each sample. The error rate of each sample was 0.03%. The Q20 of each sample was above 96%, the Q30 was above 90%, and the GC content was between 43.74% and 44.64%, indicating that the reliability of sequencing data could be used for subsequent analysis (Table S1). The reads were mapped properly against the *A. buxifolia* reference genome between 92.83% and 94.87%. Among them, the highest proportion of alignment to the unique position of the reference genome was B1 (89.73%), and the lowest was CK1 (87.76%). The proportion of alignment to multiple locations of the reference genes was between 4.6% and 5.39%, while the proportion of alignment to the positive and negative strands of the reference genome was about 43% and 44%. These results indicated that the sequencing data had a high alignment rate with the reference genome, confirming the reliability of the data (Table S2).

#### 3.7.2. Differential Gene Expression Analysis

**Figure 8:**
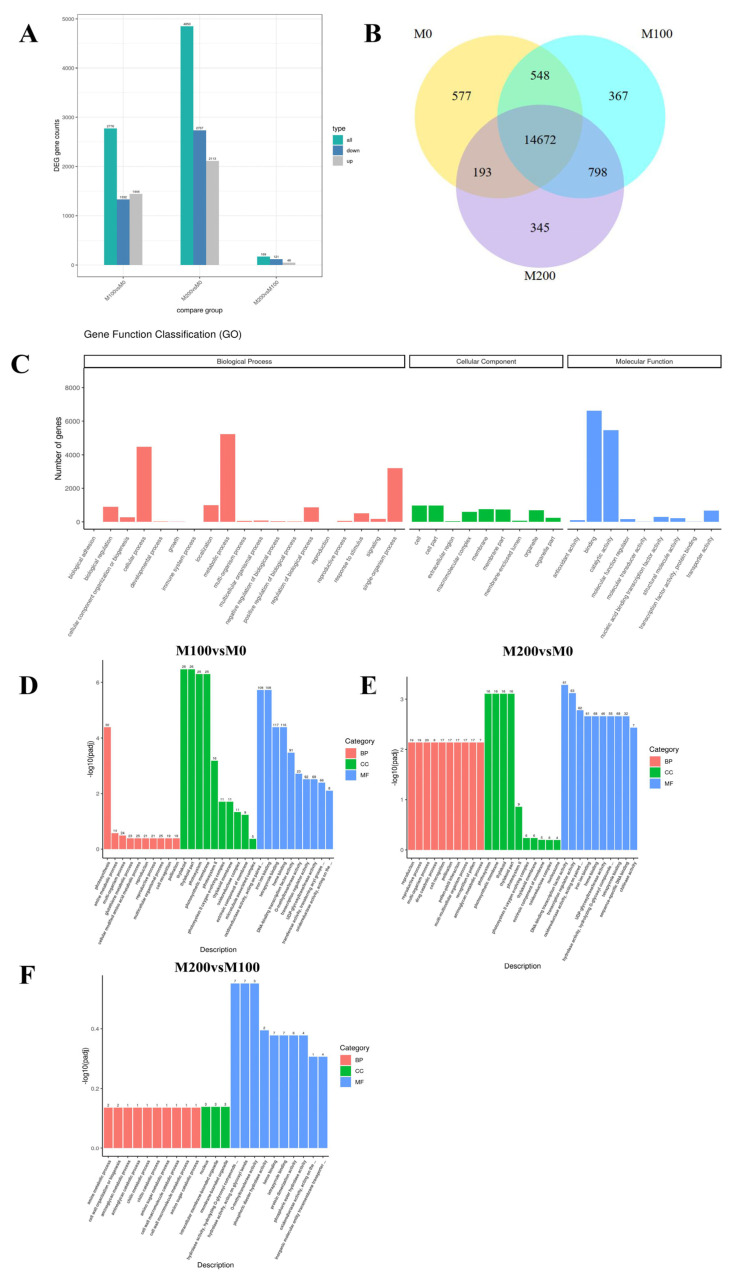
Differential gene expression analysis. (**A**) Statistics of differentially expressed genes among three comparison groups of samples (M0: under 0 mM NaCl treatment; M100: 100 mM NaCl treatment; M200: 200 mM NaCl treatment). Note: blue and gray represent up-regulated and down-regulated differential genes, respectively. The number on the column represents the number of differential genes. (**B**) Venn diagram of DEGs among the three treatments, M0, M100, and M200. (**C**) GO annotation analysis of differentially expressed genes involved in biological process, cellular components, and molecular function. (**D**–**F**) GO enrichment analysis histogram.

A total of 2776 DEGs were identified, of which 1444 were up-regulated and 1322 were down-regulated in the comparison of M100 vs. M0; a total of 4850 DEGs were identified in the comparison of M200 vs. M0, up-regulated 2113 and down-regulated 2737; there were 169 differential genes in the comparison of M200 vs. M100, up-regulated 48 genes were up-regulated and 121 down-regulated genes were down-regulated (Figure 8A).

As shown in the Venn diagram (Figure 8B), there were 14,672 co-expressed genes between the three treatments, 548 co-expressed genes between M100 and M0, 193 co-expressed genes between M200 and M0, and 798 co-expressed genes between M200 and M100. In addition, M0 treatment contained 577 unique differential genes, 367 unique differential genes were contained in M100 treatment, and 345 unique differential genes in M200 treatment (Figure 8B).

#### 3.7.3. GO Enrichment Analysis of DEGs

Compared with the Gene Ontology (GO) database, a total of 12,081 unigenes are annotated. Further classification was performed according to unigenes annotated with the three major GO classification sub-nodes (TERM), and 787 GO detailed annotations were obtained. According to all the annotations to the second level, the numbers of genes were different in biological process, cellular component, and molecular function. GO classification showed that many DEGs were enriched that were involved in metabolic processes, cellular processes, and single-organism processes in the classification of the biological process (Figure 8C). In the classification of the cellular process, many DEGS were enriched that were involved in cells, cell parts, membranes, membrane parts, and organelles (Figure 8C). In the classification of the molecular function, many DEGs were enriched that were involved in binding, catalytic activity, and transporter activity (Figure 8C).

GO functional enrichment takes padj less than 0.05 as the threshold for significant enrichment. According to the threshold, the most significant 30 terms were selected to draw a histogram. If there were less than 30 terms, all terms were drawn, as shown in Figure 8D–F. Comparison between the three different treatments showed that most of the differential expressed genes in the leaves of *A. buxifolia* seedling were enriched in biological processes and molecular functions, and a small part was in cell composition.

#### 3.7.4. KEGG Functional Enrichment Analysis of DEGs

**Figure 9:**
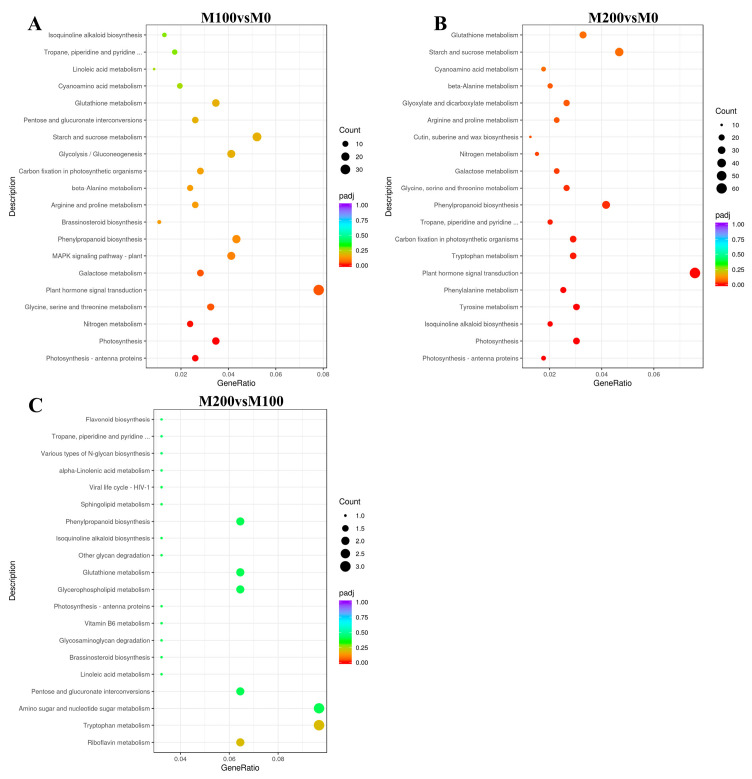
DEGs enriched KEGG pathway scatterplot; cluster heat map of DEGs. (**A**) M100vsM0; (**B**) M200vsM0; (**C**) M200vsM100.

To investigate key biological pathways of *A. buxifolia* seedling in response to salt stress, KEGG enrichment analysis was performed on these DEGs from the M0, M100, and M200 treatment. DEGs between M100 vs. M0 treatment were annotated to 111 pathways, the top 20 pathways included plant hormone signal transduction, starch and sucrose metabolism, glycolysis/gluconeogenesis signaling pathway, photosynthesis, glutathione metabolism pathway (Figure 9A). DEGs between M200 and M0 treatment were annotated to the 113 KEGG pathways and the top 20 pathways included starch and sucrose metabolism, phenylpropanoid biosynthesis pathways, glutathione metabolism pathways, and tyrosine metabolism (Figure 9B). DEGs between M200 and M100 treatment were annotated to 34 KEGG pathways, and the top 20 pathways included tryptophan metabolism pathway, amino sugar and nucleotide sugar metabolism (Figure 9C).

#### 3.7.5. Analysis of Genes Responsive to Salt Stress

**Figure 10:**
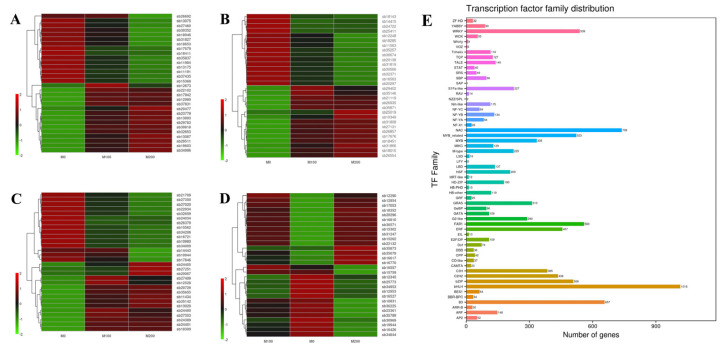
Differential gene KEGG functional enrichment analysis. (**A**) Glycerolipid metabolism; (**B**) plant hormone signal transduction; (**C**) glutathione metabolism; (**D**) starch and sucrose metabolism. Note: The abscissa in the diagram is the sample name, and the ordinate is the normalized value of the differential gene FPKM. The redder the color, the higher the expression; the greener the expression, the lower the expression. (**E**) Number of transcription factor family annotations of differential expressed genes.

After salt-stress treatment of *A. buxifolia* seedlings with different NaCl concentrations, DEGs enriched in the KEGG pathway, such as glycerolipid metabolism (*cic00561*), plant hormone signal transduction (*cic04075*), glutathione metabolism (*cic00480*), and starch and sucrose metabolism, (*cic00500*) and 15 gene IDs in up and down were selected for cluster heat map display (Figure 10A–D). Among the up-regulated genes in glycerolipid metabolism were alcohol dehydrogenase (NADP+) *AKR1A1* (*sb13087*, *sb12980*, *sb37831*), inaldo-keto reductase superfamily, diacylglycerol O-acyltransferase 2 *DGAT2* (*sb17942*), and acylglycerol lipase *MGLL* (*sb19603*); the down-regulated genes were *MGD* of 1,2-diacylglycerol 3-beta-galactosyltransferase (*sb35837*), ATS1of glycerol-3-phosphate O-acyltransferase (*sb18411*, *sb30352*, *sb26692*), and *SQD2* of sulfoquinovosyl transferase (*sb13075*) (Figure 10A). In plant hormone signal transduction, auxin responsive GH3 gene family *CH3* (*sb18451*), jasmonate ZIM domain-containing protein *JAZ* (*sb29402*, *sb35871*), and ABA responsive element binding factor *ABF* (*sb26554*, *sb31866*) were up-regulated. Down-regulated genes include *ARR-A* of ARR-A family(*sb35257*), *AUX1* of auxin influx carrierinAUX1 LAX family (*sb24722*), and *DELLA* of DELLA protein (*sb20297*, *sb32371*, *sb16563*) (Figure 10B). The up-regulated genes in glutathione metabolism included GST of glutathione S-transferase (*sb20067*, *sb24400*, *sb20729*), *GGCT* of gamma-glutamylcyclotransferase (*sb24389*), and RRM1of ribonucleoside-diphosphate reductase subunit M1(*sb35655*); the down-regulated genes included glutathione dehydrogenase/transferase *DHAR* (*sb24034*), 6-phosphogluconate dehydrogenase gntZ (*sb34206*), and gamma-glutamyltranspeptidase *GGT1* (*sb15562*) (Figure 10C). The up-regulated genes in starch and sucrose metabolic pathways included beta-fructofuranosidase sacA (*sb16770*, *sb17053*, *sb16810*), alpha-glucosidasemalZ (*sb12854*), and alpha-amylase *AMY* (*sb15302*), and the down-regulated genes included granule-bound starch synthase *WAXY* (*sb12345*), glucose-1-phosphate adenylyltransferase GlgC (*sb25773*), and glucan endo-1, 3-beta-glucosidase 5/6*GN5/6* (*sb16527*) (Figure 10D).

#### 3.7.6. Annotation Analysis on Transcription Factor

Through the transcriptome data obtained from the seedlings of *A. buxifolia* treated with different NaCl concentrations, the plant transcription factor database (PlantTFDB) in PlantTFDB (v3.0) was used for comparisons, and 10,121 gene sequences were successfully annotated to 58 transcription factor families. The most differentially expressed genes were alkaline helix-loop-helix bHLH (985 genes), NAC (692 genes), B3 (632 genes), FAR1 (545 genes), MYB-related (502 genes), WRKY (495 genes), alkaline leucine zipper bZIP (479 genes), and some transcription factor family genes such as MYB and AP2 were expressed, indicating that these gene families play a close role in the response of *A. buxifolia* seedlings to salt stress (Figure 10E).

### 3.8. Comparison of Gene Expression FoldChanges

**Figure 11:**
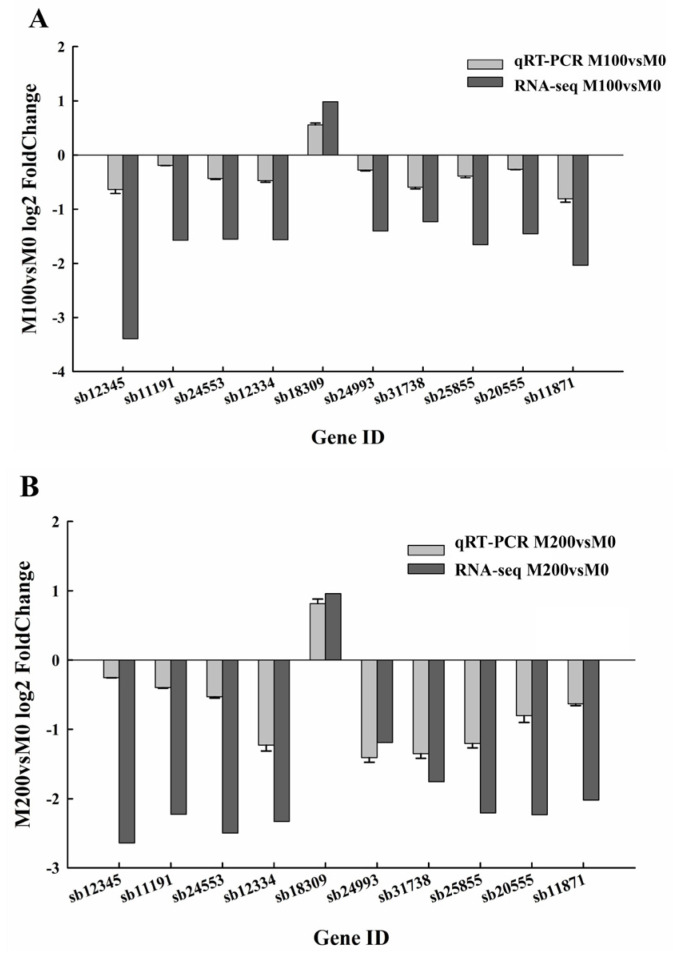
Verification of RNA-Seq results by qRT-PCR. (**A**) M100 vs. M0 log_2_ FoldChange. (**B**) M200 vs. M0 log_2_ FoldChange.

In the present study, two distinct techniques, namely quantitative real-time polymerase chain reaction (qRT-PCR) and RNA sequencing (RNA-seq), were utilized to respectively analyze the alterations in gene expression under the conditions of M100 vs. M0 and M200 vs. M0. As illustrated in Figure 11A, among the analyzed genes, *sb18309* emerged as the sole gene demonstrating positive log_2_ foldchange values across both qRT-PCR and RNA-seq methodologies. This finding strongly indicates that the expression of *sb18309* was significantly upregulated in the M100 condition relative to M0 treatment. Conversely, a majority of the remaining genes, including *sb12345*, *sb11191*, *sb24553*, *sb12334*, *sb24993*, *sb31738*, *sb25855*, *sb20555*, and *sb11871*, exhibited negative log_2_ foldchange values, suggesting a consistent pattern of down regulation in the M100 condition, compared with M0.

As shown in Figure 11B, most of the genes, including *sb12345*, *sb11191*, *sb24553*, *sb12334*, *sb24993*, *sb31738*, *sb25855*, *sb20555*, *and sb11871*, had negative log_2_ FoldChange values. This clearly reflects that, compared with M0, the expression of these genes was down-regulated under M200, that is, their transcriptional activity was inhibited. Among the genes examined, *sb18309* was the only gene with positive log_2_ FoldChange values in both detection methods. Under M200, the expression of the gene *sb18309* was upregulated, compared to M0, indicating that the transcriptional activity of this gene was enhanced in the M200 state. The data from qRT-PCR and RNA-seq generally showed similar trends, indicating that the data are accurate and reliable.

## 4. Discussion

Soil salinization has become a global problem, affecting plant growth and development, agricultural production and yield, and ecological environment. In recent years, more and more attention has been paid to the response of salt-tolerant plants to salt stress. Understanding the physiological and molecular mechanisms of plants under salt stress is helpful for genetic improvement of horticultural plants and improvement of salt tolerance. The salt tolerance mechanism of the coastal plant *A. buxifolia* has not been specifically confirmed. In this paper, salt stress was studied to understand its physiological and molecular mechanisms.

### 4.1. Effect of Salt Stress on Seed Germination and Seedling Growth of A. buxifolia

Salt stress not only affects seed germination but also affects seedling growth through osmotic stress and ion toxicity [20,32]. The germination rate of *A. buxifolia* seeds decreased with the increase in NaCl concentration (Figure 1A–D), and the relative salt damage rate also increased (Figure 1E). With the increasing NaCl concentration, the water potential around the seeds gradually decreased, which resulted in the difficulty of water absorption of *A. buxifolia* seeds under osmotic stress, resulting in the decrease in seed germination rate, and the increase in relative salt damage rate [18]. In addition, due to the lack of seed water absorption, the nutrients in the endosperm could not be effectively utilized and the respiration was inhibited, resulting in decreased seed germination and delayed germination [33]. Moreover, seeds also alleviate the damage caused by salt stress by being in a semi-dormant state [34]. In this study, compared with control, the germination rate and germination potential of *A. buxifolia* seeds did not show significant changes under low concentrations of 100 mM NaCl (Figure 1A,B), indicating that low concentration of NaCl had little effect on the seed germination and seedling growth of *A. buxifolia*, and it could adapt to the low concentration of NaCl culture environment through its own mediation. This is consistent with the results of Cai and [35] on the germination rate of 5 strains in the salt tolerance experiment of plateau quinoa seeds. In addition, the seeds in the 300 mM treatment still had a germination rate of 38.6% (Figure 1A), suggesting that the seeds still had certain vigor under such high NaCl concentrations. In the early seedling development stage, salt stress will inhibit the growth of epicotyl and radicle of seeds, and then affect the biomass after seed germination. The biomass of *A. buxifolia* seedlings decreased with the increase in salt concentration (Figure 5), which was consistent with that reported by Wang et al. [36]. Their results showed that the whole plant physiological quantity of alfalfa (*Medicago sativa* L.) was inhibited under salt stress. There was no significant change in dry weight, which may be due to the cotyledons still attached to the plant during the early development stage of *A. buxifolia* seedlings, and most of the weight of the plant after drying comes from the cotyledons.

Salt stress has an intuitive effect on plant morphology, which can be used as an important basis for measuring plant salt tolerance [37]. Salt stress can cause leaf senescence, leading to yellowing and eventually causing leaf wilting and abscission [38]. In this study, the leaves of *A. buxifolia* began to yellow under 100 mM NaCl treatment, while the leaves had withered under 300 mM NaCl treatment (Figure S1), which was similar to that reported by Abid et al. [39]. They reported that the kiwifruit of the salt-tolerant genotype showed a series of salt damage symptoms, such as yellowing, wilting, and shedding under salt stress. Previous studies showed that salt stress causes the accumulation of chloride ions in plant leaves, further triggering the synthesis of 1-aminocyclopropane-1-carboxylic acid (ACC) and ACC is efficiently converted into ethylene. When the hormone reaches a threshold, leaf abscission occurs [40]. The yellowing of leaves eventually led to a decrease in biomass (Figure 4B,E), but the fresh weight, dry weight, and relative water content showed an upward trend under 100 mM NaCl treatment. It was speculated that the seedlings of *A. buxifolia* could adapt to low NaCl concentrations, leading to decreased water potential in the seedlings and increased metabolism, which promote plant growth [18]. Our results were similar to those reported by Yan et al. [41] about NaCl treatment of the salt-tolerant shrub *Reaumuria songarica*. Plant roots are the major organ to perceive environmental stress, and this information is gradually transmitted to the above ground parts. In this study, under 300 mM NaCl treatment, except for root diameters, the significant reduction in root length, root surface areas, and root tip numbers might be related to ion toxicity (Figure 4C,D). Our results are similar to those observed by Yu [42] about salt-stress treatment of *Bolboschoenus planiculmis*. However, under 100 and 200 mM NaCl treatment, compared with control, there was no significant change in the indexes of the roots of *A. buxifolia* seedlings, indicating that there was no significant effect on the root growth of *A. buxifolia* seedlings under the two NaCl treatments. Previous studies have shown that plants adapt to salt stress by changing the cell wall structure to control cell expansion along the radial axis in the epidermis and cortex [16]. In this study, the root diameters of *A. buxifolia* seedlings showed an expansion trend, and the ground diameter was also expanded under 100 mM NaCl treatment, which may be related to the control of cell expansion direction by cell wall structure.

### 4.2. Effects of Salt Stress on Gas Exchange in A. buxifolia Seedlings

Photosynthetic pigments are an integral and vital part of the photosynthetic mechanism and play a key role in light capture and light protection. Chlorophyll and carotenoid are the main photosynthetic pigments [43]. In this study, with the increase in NaCl concentration, the levels of chlorophyll a, chlorophyll b, and total chlorophyll in the leaves of *A. buxifolia* seedlings decreased (Figure 5A), indicating that the levels of chlorophyll was greatly affected by salinity. Salt stress leads to the lack of water supply in plants, and then affects the levels of chlorophyll by destroying the biosynthesis pathway to inhibit the synthesis of chlorophyll and accelerate its degradation [44]. The decrease in chlorophyll levels further leads to the decrease in photosynthetic available area and thus affects photosynthetic efficiency. As a whole, from the perspective of photosynthetic pigments, with the increase in salt concentration, the chlorophyll content decreased and the carotenoid content increased, indicating that the increase in salt concentration affected the photosynthetic pigment content of the leaves of *A. buxifolia* seedling and further affected the photosynthesis of the plants.

Chlorophyll fluorescence is considered to be a vital tool for plant photosystem research under abiotic stress, which can reflect the effect of salt stress on photosynthesis [45]. Few chlorophyll fluorescence parameters are often detected in plants under abiotic stress. The chlorophyll fluorescent parameter, photochemical quenching coefficient (*qP*) in photosystem II (PSII) indicates that the light energy absorbed by photosynthetic pigments is used for photosynthesis, the maximum photochemical efficiency (*Fv*/*Fm*) represents the maximum efficiency of light energy conversion by photosystem II (PSII) under optimal conditions. It reflects the integrity and functional status of the PSII reaction center, including the efficiency of electron transport and the ability to capture light energy. The variable non-photochemical fluorescence quenching (*qN*) represents the degree of damage to the photosynthetic mechanism and the ability of heat dissipation, which is the self-protection mechanism in plants. With increasing NaCl concentration, *Fv*/*Fm* and qP in photosystem II (PSII) decreased significantly (Figure 5B) indicates that the PSII reaction centers were partially destroyed or inactivated by salt stress, and the electron transport chains were inhibited. This phenomenon is mainly caused by the inhibition of electron transfer from the primary receptor plastid quinone to the secondary receptor plastid quinone in the electron transport chains [46,47]. Furthermore, non-photochemical fluorescence quenching (*NPQ*) system can protect plants from photoinhibition and dissipate excess light energy as heat when plants are under abiotic stress [48]. In this study, with the increase in salt concentration, *NPQ* also showed an increasing trend. When NaCl concentration reached 200 mM, the value of *NPQ* was significantly different from that of control (Figure 5B), indicating that the leaves of *A. buxifolia* seedlings under salt stress increased heat dissipation. The way will consume too much excitation energy and improve the damage of excess light energy to the photosynthetic system by reducing the amount of light reaction. In addition, the increase in *NPQ* in plants under NaCl stress can use less energy for photosynthesis [49], which also explains the decrease in photosynthetic rate in this study. Excessive light energy absorbed by PSII can be consumed by heat dissipation to avoid inactivation and damage of photosynthetic apparatus. In this study, *qP* increased and *qN* decreased significantly (Figure 5B). It shows that the light energy absorbed by PSII is partially reduced as chemical energy, and the leaves of *A. buxifolia* seedlings dissipate the excess light energy absorbed by their own protection mechanism through heat dissipation to prevent the damage of salt stress to their own photosynthetic systems [50].

Plants can adapt to the harm caused by salt stress by regulating photosynthesis, such as regulating the assimilation efficiency of CO_2_, electron transport chains, and the distribution of photosynthetic pigments [38]. At the initial stage of salt stress, plants regulate transpiration by slowing stomatal conductance (Cond) and reducing transpiration rate (Trmmol) to reduce water loss in plants, resulting in a decrease in intercellular CO_2_ concentration (Ci) and stress response [51], leading to the decrease in photosynthetic rate in plants caused by stomatal and non-stomatal factors. In this study, in general, the net photosynthetic rate (Photo) and Ci decreased (Figure 5C,D), indicating that the decrease in Photo under salt stress was mainly caused by the decrease in Cond, which blocks the supply of CO_2_ in chloroplasts and reduces the efficiency of carbon assimilation [52]. In addition, the decrease in Cond leads to the decrease in Ci, which limits the activity of several enzymes, including ribulose-1,5-bisphosphate carboxylase (Rubisco), thereby limiting carboxylation to reduce Photo [47]. In this study, compared with control, the Cond and Temmol values decreased under 100 mM NaCl treatment, but Ci increased (Figure 5C,D), indicating that the decrease in Photo under low concentration NaCl treatment was caused by non-stomatal factors. The decrease in Photo under 100 mM NaCl treatment was not caused by CO_2_ deficiency. At this time, the accumulation of intercellular CO_2_ was caused by the decrease in intercellular CO_2_ utilization efficiency due to the decrease in chloroplast activity [52,53]. In addition, the influence of non-stomatal factors mainly is related to the inhibition of electron transport chains, the decrease in electron transfer ability of PSII donor side, and the inactivation of PSII reaction center; previous studies have shown that under stress, stomatal and non-stomatal factors and their interaction can reduce the Photo of plants [54]. In this study, it was speculated that the decrease in Photo under high NaCl concentration was caused by stomatal factors, while the decrease in Photo under low NaCl concentration treatment was caused by stomatal factors. The results reported by Ran [55] on *Salix alba* under salt stress were opposite.

### 4.3. Effects of Salt Stress on Antioxidant System of A. buxifolia Seedlings

ROS produced under salt stress causes oxidative damage to plant cells and activates their antioxidant system to scavenge excess ROS [56]. Superoxide dismutase (SOD) was the first line of defense against ROS in plant cells, which can catalyze the dismutation of the superoxide radicals into molecular oxygen and H_2_O_2_, and the produced H_2_O_2_ is effectively scavenged by catalase (CAT) and peroxidase (POD) to maintain the stability of free radicals in plant cells [57].

In general, salt stress can lead to an increase in activities of the three antioxidant enzymes, SOD, CAT, and POD, and the increase in levels of H_2_O_2_. For example, in sugar beet (*Beta vulgaris* L.) under salt stress as reported by Zhang [56], the activities of antioxidant enzymes in leaves was significantly increased, compared with the control, which was consistent with the results under high concentration NaCl treatment in this study. However, activites of SOD and CAT and levels of H_2_O_2_ in the leaves of *A. buxifolia* seedlings under 100 mM NaCl treatment were lower than those under control treatment (Figure 6A,B and Figure 7E), in which SOD activity was significantly lower. It was speculated that under low concentration of NaCl, *A. buxifolia* seedlings might be accompanied by mild stress. The plant cells regulated the osmotic pressure of Na^+^, and increased the water absorption of these seedlings, so that these seedlings were under relatively suitable conditions, resulting in no excessive production of superoxide free radicals in these seedlings. The response mechanism of SOD was not stimulated, thus SOD activities were in a low state. This result was similar to that reported by Parvin [58] about reduced SOD and CAT activities in treated tomato (*Solanum lycopersicum* L. cv. Pusa Ruby) with quercetin under salt stress. However, some researchers believed that the decrease in activities of SOD, CAT, and POD may be due to the sensitivity of plant cells to salt stress [59].

Under stress conditions, the ascorbic acid-glutathione (AsA-GSH) pathway is the major pathway to defense against oxidation, which mainly is involved in resisting oxidative stress and scavenging H_2_O_2_ in plant cells [60]. In AsA-GSH pathway, ascorbate peroxidase (APX) catalyzes the conversion of H_2_O_2_ to H_2_O by catalyzing AsA, and at the same time AsA is converted to monodehydroascorbic acid (MDHA), and then monodehydroascorbic acid reductase (MDHAR) reduces MDHA to AsA through the electrons of the electron transport chains, and spontaneously forms a part of dehydroascorbic acid (DHA) [60,61]. In this study, activities of APX (Ascorbate peroxidase) and and levels AsA (ascorbic acid) reached the highest value under 200 mM NaCl treatment, which was significantly higher than the other three treatments (Figure 6D), and CAT activities increased significantly under this treatment (Figure 6B). Although H_2_O_2_ levels under this treatment were higher than those under control and 100 mM NaCl treatment (Figure 7E), there were no significant changes, indicating that *A. buxifolia* seedlings degraded H_2_O_2_ to a certain extent through the catalysis and reduction of AsA under salt stress. The metabolite GSH (glutathione) in plant cells can directly scavenge singlet oxygen and hydroxyl radicals, and possesses the function of signal transduction. Dehydroascorbic acid (DHAR) catalyzes the reduction of DHA to AsA, at the same GSH is oxidized to oxidized glutathione (GSSG). And then GSSG is reduced to GSH by function of glutathione reductase (GR) [60,61]. As an antioxidant, GSH had a strong effect on improving stress resistance. In this study, the increase in salt concentrations showed an upward trend in GR activity, while GSH showed an upward trend when the salt concentration increased from 0 to 200 mM, and showed a downward trend when the NaCl concentration exceeded 200 mM (Figure 6C), indicating that the increase in salinity activated GR activity and increased the salt tolerance of *A. buxifolia* seedlings.

The accumulation of ROS can lead to oxidative damage in plant cells, especially biomembranes. Membrane lipid peroxidation causes leakage of inorganic ions in plant cells [3]. Malondialdehyde (MDA) was the product of lipid peroxidation of cell membrane, and conductivity of plant tissues reflects the effect of salt stress on cell membrane permeability, which was an important indicator of oxidative stress response of plants under environmental stress. Therefore, the levels of MDA (malondialdehyde) and relative electrolyte leakage (REL) were proportional to the severity of lipid peroxidation and membrane damage [62]. Compared with control, there was no significant difference in levels of MDA and REL under 100 mM NaCl treatment (Figure 7C,D), indicating that under 100 mM NaCl treatment, the leaves of *A. buxifolia* seedlings were less damaged by oxidative stress and the degree of cell membrane damage was less. The cell membrane system was in the first part of the salt-stress damage, indicating better stability of the cell membranes under this treatment [5]. In addition, MDA and REL levels of *A. buxifolia* seedlings increased significantly under high concentration NaCl treatment, indicating that salt stress caused strong lipid peroxidation of cell membranes, and the function of cell membrane antioxidant system was overcome, resulting in serious damage to cell membranes, thus affecting the physiological metabolism of *A. buxifolia* seedlings [63].

### 4.4. Effects of Salt Stress on Osmotic Adjustment System of A. buxifolia Seedlings

Plants maintain the structures of proteins and the stability of cell membrane through the accumulation of osmotic substances, such as soluble sugars (SS), soluble proteins (SP), and proline, which reduce cell osmotic pressure to improve the ability of plants to resist salt stress [58]. The accumulation of proline is helpful to the stability of cell membrane and reduces the adverse effects of NaCl on cell membrane damage. In this study, with increase in NaCl concentration, the proline levels of *A. buxifolia* seedlings showed a significant increase to alleviate the harm caused by osmotic stress (Figure 7B), which is consistent with the results obtained on of three citrus rootstocks under salt tolerance [64] and *Juglans macrocarpa* [63]. Under salt stress, SP played a key role in osmotic regulation and maintenance of membrane integrity [11]. In this study, the levels SP of *A. buxifolia* seedlings increased with the increase in NaCl concentration (Figure 7A), indicating that *A. buxifolia* seedlings could improve the adaptability of saline environment and reduce the damage of salt stress to plant cells by regulating osmotic potential. SS can regulate the change in cell osmotic potential, which is the carbon framework in the cells and an important energy source for plants. In this study, SS levels of *A. buxifolia* seedlings showed an increasing trend (Figure 7A), but under 100 mM NaCl treatment, the SS levels were at the lowest value, which may be due to the fact that the *A. buxifolia* seedlings were under suitable conditions without too much SS to resist osmotic stress, or may be due to the significant increase in proline levels and SP under this treatment or the influence of other osmotic adjustment species to alleviate osmotic stress [65].

### 4.5. Effects of Salt Stress on Transcriptome Analysis of A. buxifolia Seedlings

Plant salt tolerance is a complex and comprehensive process involved in multiple genes and multiple metabolism pathways [6]. Expression analysis GO differential genes was mainly enriched in metabolic processes, cellular processes, cells, cell composition, binding and catalytic activity, etc., and with the increase in NaCl concentration, the expression of salt tolerance-related genes increased, including signal transduction, photosynthesis, lipid metabolism, carbohydrate metabolism, protein metabolism, amino acid metabolism, active transmembrane transporter activity, and cell membranes, etc. Some DEGs were related to plant cell membrane. The plant membrane system was the first site to perceive salt stress. The accumulation of ROS under salt stress leads to cell damage and further results in membrane lipid peroxidation. This explains that a large number of entries in the GO enrichment analysis of salt tolerance of *A. buxifolia* seedlings were related to cell membranes.

The response of plants to salt stress was related to the results of KEGG functional enrichment for DEGs. The results showed that the differentially expressed genes in the three groups were mainly glutathione metabolism (*cic00480*) and photosynthesis-antenna proteins (*cic00196*), while M200 vs. M0 and M100 vs. M0 had a variety of similar enrichments, such as plant hormone signal transduction (cic04075), starch and sucrose metabolism, and photosynthesis. Glutathione S-transferase (GST) family genes, including *sb22934*, *sb20067*, and *sb20729*, play an important role in plant antioxidant pathways. The enzymes encoded by these genes are involved in peroxide detoxification [66]. Furthermore, 17 related genes were down-regulated in the photosynthesis-antenna protein pathway, indicating that the *A. buxifolia* seedlings may reduce the absorption of light energy through the light capture complex to protect the *A. buxifolia* plants to adapt to the salt stress, which was similar to the KEGG functional enrichment reported by Lu [67] on grapes under salt stress. It is shown that under NaCl stress, *A. buxifolia* seedlings reduced the absorption of chlorophyll to light energy by down-regulating related genes, so as to reduce the damage of excessive accumulation of light energy to photosynthetic system. In addition, auxin (IAA), abscisic acid (ABA), jasmonic acid (JA), brassinosteroids (BRs), and ethylene (ETH) play important roles in plant salt stress and defense [66]. The response genes involved in the IAA pathway mainly include auxin response proteins (Aux/IAA), auxin response factors (ARF), auxin response proteins (SAUR), and GH3. In this study, these genes are all involved in corresponding response regulation under salt stress, which is similar to the expression of genes involved in IAA pathway in citrus roots after salt stress, as reported by Xie [67]. The up-regulation and down-regulation of Aux/IAA, SAUR, and GH3 genes reflect that salt stress may induce IAA to regulate *A. buxifolia* seedlings to increase the stress resistance of plants [68]. Furthermore, ABA plays an important role in plant resistance to abiotic stress. Under salt stress, the genes related to serine/threonine protein kinase SIK2 (SnRK2) and protein phosphatase 2C (PP2C) in the ABA pathway of *A. buxifolia* seedlings were up-regulated, while the ABA receptor (PYR/PYL) gene was down-regulated, and ABA response element binding factor (ABF) gene was up-regulated and down-regulated, indicating that PYR/PYL and PP2C were regulated, resulting in the accumulation of SnRK2 and the phosphorylation of downstream substrates [69]. This was similar to the results reported by Lu [67] on ABA pathway of grape and those reported by Xie [70] after salt stress, which indicates that the expression of these genes under salt stress changes the levels of ABA to reduce the damage of salt stress to the seedlings.

Previous studies showed that the TFs family played important roles in plant resistance to salt stress, such as bHLH, NAC, WRKY, MYB, and bZIP [71]. In this study, DEGs obtained by transcriptome sequencing under salt stress were annotated to 45 TFs families. It was speculated that these family transcription factors play important roles in resistance of *A. buxifolia* seedlings to salt stress, such as bHLH family, NAC family, WRKY family, MYB-related family, bZIP family and so on. bHLH is an important TF family with basic/helical loop helix structure, which was involved in the response of many plants to abiotic stress. Previous studies showed that the transient expression of *CabHLH035* enhances the salt tolerance of pepper, while the ectopic expression of *CabHLH035* enhances the salt tolerance of *Arabidopsis thaliana* [72]; NAC (NAM, CUC and ATAF) TF family was one of the unique TFs family in plants. Previous studies showed that NAC family genes play important roles in resisting abiotic stress. Wang et al. [73] constructed the transgenic *A. thaliana* lines of potato *StNAC053* gene. Their results showed that the tolerance of *StNAC053* transgenic Arabidopsis lines to salt stress and drought stress was significantly increased, and the expression levels of several stress-related genes, including *COR15A*, *DREB1A*, *ERD11*, *RAB18*, *ERF5*, and *KAT2* were up-regulated. The WRKY family is widely involved in plant growth and development, defense regulation, and stress response. In salt-stress response, MAPK genes can act as crosstalk nodes between ABA signaling and other signaling pathways to regulate the expression of genes involved in metabolism pathways and related genes to participate in salt-stress response [74]. Since the first plant MYB-related gene (*MybSt1*) was isolated from potato, many MYB-related family genes were identified to play key roles in the development and stress of transcription regulators [75]. bZIP family has the functions of regulating plant flower development, seed maturation, pathogen defense, and stress signal transduction. It was confirmed in different plants that TFs encoded by bZIP genes can enhance the tolerance of plants to salt stress. For example, Kang et al. [76] cloned gene *IbbZIP1* in the HVB-3 series sweet potato [*Ipomoea batatas* (L.) Lam.]. Zhao et al. [77] cloned the gene *BnaABF2* in rapeseed (*Brassica napus* L.). The two genes were overexpressed in *Arabidopsis thaliana* to enhance its salt tolerance. In addition, transcription factors such as AP2, ERF, B3 [78], and C2H2 [79], were found in this study. These transcription factor family genes were confirmed to play a crucial role in salt stress. Therefore, the annotated transcription factor family in *A. buxifolia* seedlings may play an important regulatory role in the response of *A. buxifolia* seedlings to salt stress. In summary, the transcription factors identified in *A. buxifolia* do not function in isolation but are likely integrated into a coordinated regulatory network. Among them, members of the NAC and bHLH families may serve as upstream regulatory hubs, perceiving salt stress signals and initiating early transcriptional reprogramming. Meanwhile, transcription factors from the WRKY, MYB, and bZIP families may act as relay nodes, transmitting and refining these signals to specifically activate downstream functional genes, such as those encoding ion transporters, osmolyte biosynthetic enzymes, and antioxidant enzymes, thereby collectively shaping the salt-stress-tolerant phenotype of *A. buxifolia* seedlings.

In this study, qRT-PCR was performed to validate the RNA-Seq results. Among the analyzed genes, *sb18309* emerged as the sole gene showing positive log_2_ fold-change values in both qRT-PCR and RNA-seq analyses. The *sb18309* gene in *A. buxifolia* encodes a putative glutathione S-transferase (GST), bioinformatically classified as a Phi-class (GSTF) enzyme based on significant sequence similarity to the canonical Phi-class GST from *Hyoscyamus muticus* (UniProt P46423) [80].The predicted protein product exhibits the conserved domain architecture characteristic of cytosolic GSTs, comprising an N-terminal thioredoxin-like domain (PF02798) housing the glutathione-binding site (G-site) and a C-terminal α-helical domain (PF00043) forming the hydrophobic substrate-binding site (H-site). This domain organization indicates a biochemical function in catalyzing the conjugation of glutathione (GSH) to electrophilic xenobiotics (e.g., environmental toxins) and endogenous metabolites, thereby enhancing their solubility for vacuolar sequestration or cellular efflux [81]. Consequently, the *sb18309* gene product is predicted to function in detoxification pathways and enhance oxidative stress tolerance, providing cellular protection against biotic and abiotic stressors [82,83].

## 5. Conclusions

In summary, salt-stress inhibited seed germination and seedling growth of *A. buxifolia*. Compared with control, there were no significant changes in the germination rate, germination potential, relative salt injury rate, biomass and relative water content of the seeds and the growth, enzyme activity and lipid peroxidation products of the seedlings of *A. buxifolia* under 100 mM NaCl treatment indicating that the lower salinity concentration did not affect germination. Under 300 mM NaCl treatment, the seeds and seedlings of *A. buxifolia* showed certain vitality, which indicated that *A. buxifolia* survived to some extent under such high NaCl concentrations. In addition, according to the transcriptome analysis, different concentrations of NaCl treatment can induce the expression of related genes to resist the harm of salt stress. Consequently, the findings indicate that the tolerance of this species to 100 mM NaCl makes it suitable as experimental material for investigating salt tolerance mechanisms and a candidate for developing salt-tolerant citrus rootstocks.

## Data Availability

The data presented in this study are available on request from the corresponding author.

## Supplementary Materials

The following supporting information can be downloaded at: https://www.mdpi.com/article/10.3390/biology15010065/s1, Figure S1: Effects of different NaCl concentrations on the characteristics of A. buxifolia seedlings (Bar = 1 cm); Table S1: Quality Statistics of sequencing data; Table S2: Comparison statistics of sequencing data and assembly results.
