# Supplementary material for: Response of Atalantia buxifolia to Salt Stress Based on Physiological and Transcriptome Analysis
Source: Biology (Basel). 2025 Dec 30;15(1):65. doi: 10.3390/biology15010065 (PMC12784857; doi:10.3390/biology15010065)
Supplement: Supplementary file 1 [file biology-15-00065-s001.zip › biology-3994369-supplementary.pdf]

Supplementary Figures

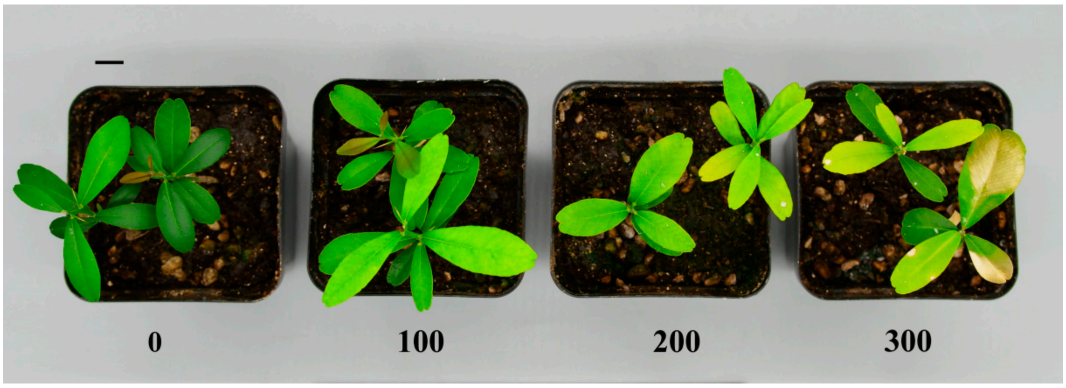

Figure S1 Effects of different NaCl concentrations on the characteristics of *A. buxifolia* seedlings (Bar = 1 cm)

Table S1 Quality Statistics of sequencing data

Sample	Raw reads	Clean reads	Clean bases	Error rate	Q20 (%)	Q30 (%)	GC pct (%)
CK1	40061068	39559074	5.93G	0.03	96.36	90.51	44.27
CK2	46741806	45276126	6.79G	0.03	97.73	93.34	44.21
CK3	44092462	43697476	6.55G	0.03	96.58	90.97	44.64
J1	46711766	46087350	6.91G	0.03	96.48	90.74	44.4
J2	46610066	45933212	6.89G	0.03	96.54	90.86	44.01
J3	43395874	42657722	6.4G	0.03	96.52	90.82	43.9
B1	47133642	45216058	6.78G	0.03	96.88	91.52	44.31
B2	46262562	45793922	6.87G	0.03	96.85	91.51	44.02
B3	41632442	41194264	6.18G	0.03	96.72	91.25	43.74

Table S2 Comparison statistics of sequencing data and assembly results

Sample	Totalreads	Totalmap	Uniquemap	Multimap	Positivemap	Negativemap
CK1	39559074	36722509 (92.83%)	34715894 (87.76%)	2006615 (5.07%)	17315227 (43.77%)	17400667 (43.99%)
CK2	45276126	42953564 (94.87%)	40511416 (89.48%)	2442148 (5.39%)	20214225 (44.65%)	20297191 (44.83%)

CK3	43697476	41073999 (94.0%)	38755879 (88.69%)	2318120 (5.3%)	19324689 (44.22%)	19431190 (44.47%)
J1	46087350	43270961 (93.89%)	40984174 (88.93%)	2286787 (4.96%)	20451130 (44.37%)	20533044 (44.55%)
J2	45933212	43095301 (93.82%)	40912816 (89.07%)	2182485 (4.75%)	20417799 (44.45%)	20495017 (44.62%)
J3	42657722	40032813 (93.85%)	38072396 (89.25%)	1960417 (4.6%)	19006962 (44.56%)	19065434 (44.69%)
B1	45216058	42786679 (94.63%)	40572778 (89.73%)	2213901 (4.9%)	20248806 (44.78%)	20323972 (44.95%)
B2	45793922	43253225 (94.45%)	41052094 (89.65%)	2201131 (4.81%)	20498499 (44.76%)	20553595 (44.88%)
B3	41194264	38662799 (93.85%)	36749529 (89.21%)	1913270 (4.64%)	18347323 (44.54%)	18402206 (44.67%)

Note : Sample: sample name; Total reads: the number of clean reads after quality control of sequencing data; Total map: the number and percentage of reads aligned to the genome; Unique map: the number and percentage of reads aligned to the unique position of the reference genome (for subsequent quantitative data analysis of reads); Multi map: the number and percentage of reads aligned to multiple locations of the reference genome; Positive map: the number and percentage of reads aligned to the positive strand of the reference genome; Negative map: The number and percentage of reads aligned to the negative strand of the reference genome.
